# Innate immunity changes in soccer players after whole-body cryotherapy

**DOI:** 10.1186/s13102-022-00578-z

**Published:** 2022-10-25

**Authors:** Valentina Selleri, Marco Mattioli, Domenico Lo Tartaro, Annamaria Paolini, Giada Zanini, Anna De Gaetano, Roberta D’Alisera, Laura Roli, Alessandra Melegari, Pasqualino Maietta, Ferdinando Tripi, Emanuele Guerra, Johanna Chester, Gustavo Savino, Tommaso Trenti, Andrea Cossarizza, Anna Vittoria Mattioli, Marcello Pinti, Milena Nasi

**Affiliations:** 1 Department of Life Sciences, University of Modena and Reggio Emilia, via Campi, 287, 41125 Modena, Italy; 2 National Institute for Cardiovascular Research - INRC, 40126 Bologna, Italy; 3 Department of Medical and Surgical Sciences for Children and Adults, University of Modena and Reggio Emilia, 41125 Modena, Italy; 4 Department of Biomedical, Metabolic and Neural Sciences, University of Modena and Reggio Emilia, 41125 Modena, Italy; 5 Department of Public Healthcare, Sports Medicine Service, Azienda USL of Modena, 41121 Modena, Italy; 6 Department of Laboratory Medicine and Pathology, Azienda USL of Modena, 41121 Modena, Italy; 7 Department of Quality of Life, “Alma Mater Studiorum”, 40126 Bologna, Italy; 8 “La Fratellanza 1874” Not-for-profit sport Association, 41126 Modena, Italy; 9 Department of Surgery, Medicine, Dentistry and Morphological Sciences, University of Modena and Reggio Emilia, 41125 Modena, Italy

**Keywords:** Cytokines, Inflammation, Monocytes, Systemic cryotherapy, Soccer players

## Abstract

Whole-body cryotherapy (WBC) consists of short exposure (up to 2–3 min) to dry air at cryogenic temperatures (up to -190 °C) and has recently been applied for muscle recovery after injury to reduce the inflammation process. We aimed to determine the impact of cryotherapy on immunological, hormonal, and metabolic responses in non-professional soccer players (NPSPs). Nine male NPSPs (age: 20 ± 2 years) who trained regularly over 5 consecutive days, immediately before and after each training session, were subjected to WBC treatment (WBC-t). Blood samples were collected for the evaluation of fifty analytes including hematologic parameters, serum chemistry, and hormone profiles. Monocytes phenotyping (Mo) was performed and plasmatic markers, usually increased during inflammation [CCL2, IL-18, free mitochondrial (mt)DNA] or with anti-inflammatory effects (IL2RA, IL1RN), were quantified. After WBC-t, we observed reduced levels of ferritin, mean corpuscular hemoglobin, mean platelet volume, testosterone, and estradiol, which however remain within the normal ranges. The percentage of the total, intermediates and non-classical Mo increased, while classical Mo decreased. CXCR4 expression decreased in each Mo subset. Plasma IL18 and IL2RA levels decreased, while IL1RN only exhibited a tendency to decrease and CCL2 showed a tendency to increase. Circulating mtDNA levels were not altered following WBC-t. The differences observed in monocyte subsets after WBC-t may be attributable to their redistribution into the surrounding tissue. Moreover, the decrease of CXCR4 in Mo subpopulations could be coherent with their differentiation process. Thus, WBC through yet unknown mechanisms could promote their differentiation having a role in tissue repair.

**Supplementary Information:**

The online version contains supplementary material available at 10.1186/s13102-022-00578-z.

## Introduction

​​Soccer has been described as a high-intensity sport in which periodic aerobic and anaerobic physical actions are combined [1]. Both training and competitions induce physiological changes, which must be evaluated to better understand ideal training regimes [2]. Soccer players are exposed to different types of trauma and concussion, especially during matches, and the most frequently injured body sites seem to be knee joints and the thighs [3]. Moreover, players with ten or more episodes of concussions are at significantly higher risk of stroke, independent of cardiovascular disease and age [4]. In addition, as players intentionally use their heads to hit the ball, soccer is considered one of the sports with the highest number of repetitive head impacts (RHI). As RHI does not usually result in acute symptoms, it is described as a “subconcussive” head impact, which can generate cumulative effects [5]. Given its potential disruption to brain processes, RHI have been considered dangerous, especially for young athletes. Therefore, reduction of trauma and systemic inflammation is an important goal in improving performance.

Besides causing traumas, heavy physical loads of soccer players, particularly during the pre-season has been shown to impair immune functions. Significant reductions of absolute neutrophil and monocyte count have been observed in male soccer players after two weeks of pre-season intensive training, along with the increase pro-inflammatory markers, such as C-reactive protein, IL-1β, IL-6 and TNF-α [6]. Interestingly, the two-week tapering period following the intensive training did not lead to a complete recovery of impaired immune functions [6]. A similar increase in pro-inflammatory parameters, such as IL-1β and IL-6 has been observed in female soccer players after training [7]. In contrast with these findings, an increase in eosinophils and monocytes count has been observed after an entire season (i.e. a pre-season training followed by 12 weeks) in collegiate soccer players [8], and a positive correlation between cumulative match time -that is, time played in a season – and monocyte count of professional soccer players [9].

Soccer is a discipline with multiple games with short turnarounds. This lack of recovery time can determine a risk of insufficient recovery from trauma, inflammation and immune system alterations. Thus, several recovery strategies intended to restore the neuromuscular system have been implemented in the last years. Whole-body cryotherapy (WBC) consists of the short exposure (up to 2–3 min) to dry air at cryogenic temperatures (-110 to -195 °C) and has recently been practiced after injury for muscle recovery to reduce the inflammation process resulting from muscle overuse [10]. Such temperatures induce peripheral vasoconstriction, blood pressure increase and reduction of sympathetic nerve activity [11]. Studies on non-athletes and on rugby players demonstrated that at least two minutes of exposure are needed to significantly reduce skin temperature [12, 13]. Due to its anti-inflammatory effects, WBC has been extended for use in clinical pathologies involving systemic inflammation, such as rheumatoid arthritis, fibromyalgia, or ankylosing spondylitis [14, 15]. WBC has been shown to improve enzyme recovery response in muscles [16] and improve self-perception of recovery [17]. For these reasons, the use of WBC has gained increased popularity as recovery intervention amongst soccer players and is now commonplace in elite soccer [18]. Recent studies suggest that one or more WBC treatment (WBC-t) bout can induce acute anti-inflammatory, hormonal and physiological responses, but they have been only included limited hormonal and hematological parameters, with very few cytokines and no monocytes evaluated [10].

Several studies have underlined the importance of monitoring hematocrit (Ht) and hemoglobin (Hb) levels among hematological markers, as they seem to be important indicators of body fluid adaptation to different types of training [19]. Further, hormones are important biomarkers to detect physical performance changes induced by long-term soccer training; especially the Testosterone/Cortisol (T/C) ratio because testosterone and cortisol appear to be sensitive to the intensity of soccer training and associated fatigue [19].

Moreover, biochemical markers, such as C-reactive protein (CRP), and muscle damage markers, such as lactate dehydrogenase (LDH) and creatine kinase (CK), have been used to assess and monitor inflammation caused by long periods of soccer training [19].

Despite widely used by soccer players, there is currently a lack of research investigating the impact of WBC on innate and acquired immunity. In our previous study performed on cyclists and runners, we noticed a redistribution of the three main monocytes subsets (classical, intermediate, non-classical) during inflammation. Moreover, we found changes in the expression of cytokines and chemokines which are usually increased in this condition, such as C-C Motif Chemokine Ligand 2 (CCL2) and interleukin (IL)-18, or with anti-inflammatory effects as interleukin (IL)-2Receptor Antagonist (RA) and interleukin (IL)-1RA [20].

In an attempt to clarify the effects of WBC on innate immune response, we analyzed the effects induced by 5-once-a day sessions of WBC on a variety of inflammatory markers and immunological parameters, including hormone profile, hematologic parameters, and serum chemistry, in non-professional male soccer players (NPSPs) during a period of training. We also analysed the effect of WBC on the amount of circulating mitochondrial DNA, a potential marker of cell damage and concussions [21, 22].

## Materials and methods

### Subjects

Nine male NPSPs (age: 20 ± 2 years) were enrolled from the same soccer team by the Sports Medicine Service of Modena and they performed evening-based soccer training sessions. All the enrolled athletes have obtained a certificate of competitive soccer activity from the Sports Medicine Service. That certificate is issued after physical examination, medical history evaluation, an ECG at rest and after a stress exercise test. The only exclusion criteria were the presence of inflammatory diseases or injuries occurred just before or during the study.

This study was performed in agreement with ethical recommendations of the Declaration of Helsinki, and the Ethics Committee of Area Vasta Emilia Nord approved all experiments (protocol number 88/2018/SPER/AUSLMO). Moreover, all the participants read and signed an informed consent.

### Design

**Fig. 1:**
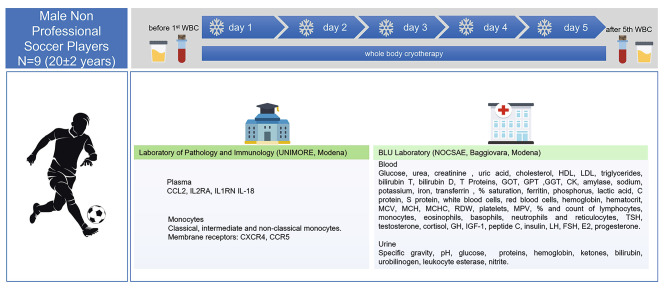
**Design of the study.** Schematic representation of the protocol. Free icons used in the figure are made by https://www.flaticon.com/authors/smashicons

This observational study provides five consecutive WBC-t sessions, administered at the mornings. WBC-t was performed in a Cryomed chamber (Cryomed Italy, Milan, Italy) and consisted of short exposure (up to 3 min) to extremely cold air (-190° C) inside a chamber in which the subject’s hands and head remain outside, therefore not in contact with the cold stimulus. Subjects were dressed in underwear avoiding the presence of metal pieces. A sample of 40 mL of venous blood was collected from each subject before the first session of WBC-t (day 1) and promptly following the fifth and final WBC-t (day 5; Fig. 1).

### Clinical chemistry, hematology and hormonal analyses

NPSPs blood and urine samples were collected before and after WBC-t and subjected to several laboratory analyses. Clinical chemistry, hematology, hormonal parameters, and urine were evaluated at the BLU Laboratory (NOCSAE, Baggiovara, Modena, certification #ISO90012015) according to hospital protocol. Regarding blood samples, the BLU Laboratory of Baggiovara evaluated a full set of fifty analytes, involving glucose, urea, creatinine, uric acid, cholesterol, high-density lipoprotein (HDL), low-density lipoprotein (LDL), triglycerides, bilirubin T, bilirubin D, T proteins, glutamic oxaloacetic transaminase (GOT), pyruvic glutamic transaminase (GPT), gamma-glutamil transferase (GGT), CK, amylase, sodium, potassium, iron, transferrin, % saturation, ferritin, phosphorus, lactic acid, C protein, S protein, white blood cells, red blood cells, hemoglobin, hematocrit, mean corpuscular volume (MCV), mean corpuscular hemoglobin (MCH), mean corpuscular hemoglobin concentration (MCHC), red cell distribution width (RDW), platelets, mean platelet volume (MPV), % and count of lymphocytes, monocytes, eosinophils, basophils, neutrophils and reticulocytes, thyroid stimulating hormone (TSH), T, C, growth hormone (GH), insulin growth factor (IGF)-1, peptide C, insulin, luteinizing hormone (LH), follicle-stimulating hormone (FSH), estradiol (E2), and progesterone. Clinical chemistry analytes were measured in the CoreLab on full-automated clinical chemistry platforms, based on state-of-the-art enzyme kinetic techniques, immunoturbidimetric techniques, colorimetric methods (Chemistry analyzerS Olympus 680 and LX20, Beckman Coulter, Brea, CA, USA). Complete blood count with formula was performed with Accucount technology for red, white cells and platelets and VCS technology, triple impedance counting, for leukocyte formula and spectrophotometric determination of hemoglobin, and reticulocytes (after staining with methylene blue) (Hematology Analyzers DXH 750 and DXH 800, Beckman Coulter, Brea, CA, USA). Blood circulating hormones were detected on full automated platforms based on CMIA methods (Chemiluminescent Immunoassay Analyzer: Architect, Abbott Laboratories, Chicago, Illinois; USA; DXI, Beckman Coulter, Brea, CA, USA; LiaisonXL, DiaSorin, Saluggia, Italy).”

Urine analysis evaluated specific gravity, pH, glucose, proteins, hemoglobin, ketones, bilirubin, urobilinogen, leukocyte esterase and nitrite. Urine analysis was made using dipstick and image capture (Urine Microscopy System IQ200 sprint connected to the Urine Chemistry Analyzer iChem Velocity, Beckman Coulter, Brea, CA, USA).

### Blood processing and plasma analysis

Peripheral blood mononuclear cells (PBMCs) and plasma were isolated in our laboratory at the University of Modena and Reggio Emilia from venous blood using a density-gradient centrifugation standard method. Viable PBMCs were stored in liquid nitrogen and plasma was stored at -80 °C until use. Plasma analysis included four soluble factors (CCL-2, IL-2RA, IL-1RN and IL-18), DNA extraction and subsequent quantification of circulating mitochondrial (mt)DNA through droplet digital PCR (ddPCR). DNA was isolated from plasma samples with the QIAmp DNA Minikit, (Qiagen, Alameda, CA, USA), in accordance with the manufacturer’s instructions. MtDNA was quantified on a Bio-Rad QX200 ddPCR droplet system, by using the ddPCR Supermix for Probes, and ddPCR assay for the mtDNA gene ND2 (1 uL; UniqueAssayID: dHsaCPE5043508) and for the nuclear gene EIF2C1 (1 uL; UniqueAssayID: dHsaCP2500349). Reagents were from Bio-Rad, Hercules, CA, USA.

The four soluble factors CCL2, IL-18, IL-2RA and IL-1RA were analyzed from plasma samples by using Ella assays (Bio-Techne, MN, USA) following the manufacturer’s instructions [23].

## Monocytes phenotyping

Moreover, a minimum of three million PBMCs were thawed and stained with the probe Aqua Live Dead (Thermo Fisher Scientific, Waltham, MA, USA) and the fluorochrome-conjugated monoclonal antibodies anti-CD16 AF488, anti-CD14 APC, anti-HLA-DR PE-Cy7, anti-CCR2 BV605, anti-CXCR4 PE and anti-CCR5 BV421 (BioLegend, San Diego, USA). Samples were acquired on an Attune Nxt flow cytometer (Thermo Fisher Scientific, Waltham, MA, USA) and data analyzed by using FlowJo 9.9.6 (Ashland, OR, USA). As previously described, we applied a sequential gating strategy to identify the three main monocytes subsets (classical, non-classical, intermediate) [10].

### Statistical analysis

Pre- and post-WBC-t quantitative variables were compared with the Wilcoxon matched-pairs signed-rank test or two-way ANOVA and Sidak’s multiple comparisons test. Correlations between molecular and clinical data were explored with linear regression analysis. Tables reported mean and standard deviation values. Column graphs represent all data with mean $\pm$standard error (SEM). Prism 8.0 (GraphPad Software Inc., La Jolla, USA) was used for statistical analyses. P < 0.05 was considered statistically significant.

## Results

### Hematological parameters, serum chemical composition and hormone profile

**Fig. 2:**
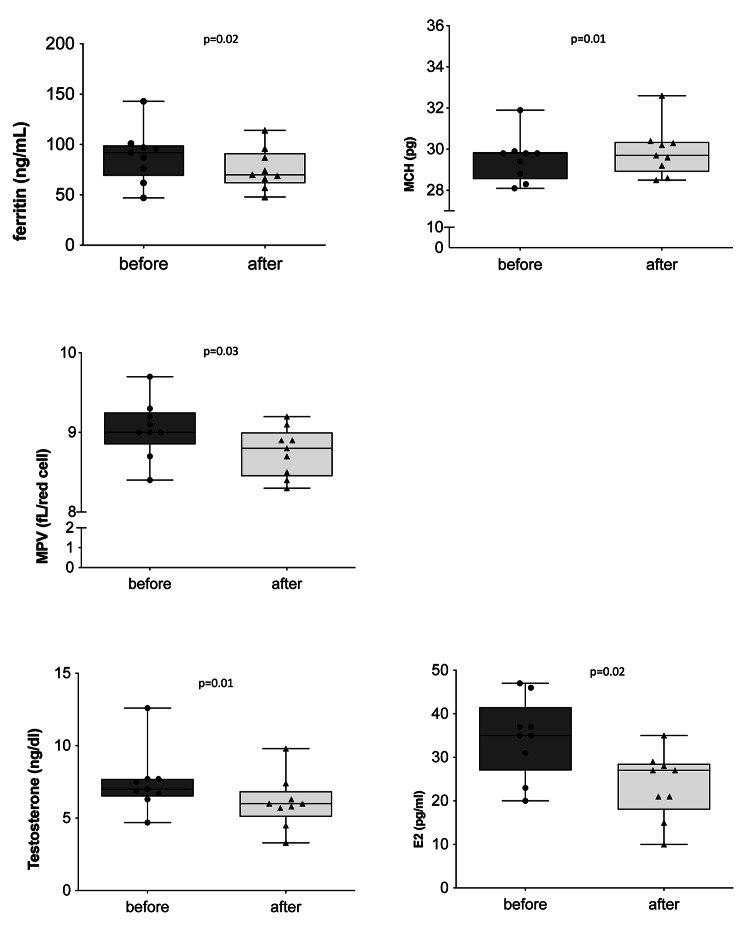
**Hematological and hormonal parameters.** Evaluation of hematological parameters levels, such as ferritin, MCH and MPV before and after five one-a-day sessions of WBC (rows 1,2). Levels of testosterone and E2 before and after five one-a-day sessions of WBC (row 3). Figures showing the single values in a box and whiskers plot. E2 indicates estradiol; WBC, whole-body cryotherapy; MCH, mean corpuscular hemoglobin; MPV, mean platelet volume

We first evaluated the effect of WBC-t on a series of hematochemical parameters normally monitored in athletes.Table 1 reports the mean and the standard deviation of all these parameters and the p value calculated by the Wilcoxon matched-pairs signed-rank test. Supplementary Table 1 reports the single values for each subject of the abovementioned parameters. All the clinical results were within normal ranges. Of the fifty blood parameters analyzed, we did not observe differences in blood glucose, urea, T proteins or phosphorus. A slight, not significant decrease of creatinine was observed among NPSPs. Urine tests did not show appreciable variations (data not shown). Twenty-four hematochemical parameters were analyzed, and we observed reduced levels of ferritin, MCH and MPV following WBC-t, as shown in the Fig. 1. Finally, we measured the levels of eleven hormones, and we found a significant decrease of testosterone and E2 (Fig. 2).

**Table 1:** **Hematochemical parameters determinations.** Evaluation of fifty hematochemical parameters normally monitored in athletes, before (T0) and after (T1) five once-a-day sessions of WBC-t. Values are expressed as mean and standard deviation, p values, calculated by Wilcoxon matched-pairs signed-rank test, were considered significant when < 0.05 (bold)

	T0				T1				
	**Mean**		**St.dev**		**Mean**		**St.dev**		**p value**
**Hematological parameters**									
White blood cells (*10^3^/µl)	6.73		1.16		6.69		0.98		0.98
Red blood cells (*10^3^/µl)	5.25		0.23		5.16		0.27		0.16
Hemoglobin (g/dl)	15.43		0.43		15.40		0.52		0.99
Hematocrit (%)	51.30		14.32		46.37		1.53		0.16
MCV (fl.)	89.40		2.89		89.96		2.89		0.43
**MCH (pg)**	29.53		1.12		29.90		1.23		**0.01**
MCHC (g/dl)	33.00		0.50		33.22		0.43		0.36
RDW (cv%)	14.01		0.78		13.92		0.61		0.45
Platelets (*10^3^/µl)	3.36		5.45		32.24		32.27		0.89
**MPV (fl.)**	9.04		0.36		8.76		0.31		**0.03**
Neutrophils %	51.37		8.16		52.48		8.00		0.45
Lymphocytes %	37.14		6.93		36.00		6.62		0.55
Monocytes %	7.73		1.38		7.62		1.38		0.51
Eosinophils %	3.19		1.82		3.34		1.55		0.58
Basophils %	0.60		0.21		0.51		0.21		0.25
Neutrofili (*10^3^/µl)	3.49		1.08		3.52		0.85		> 0.99
Lymphocytes (*10^3^/µl)	2.46		0.46		2.39		0.50		0.52
Monocytes (*10^3^/µl)	0.52		0.14		0.51		0.10		0.71
Basophils (*10^3^/µl)	0.04		0.02		0.04		0.02		0.50
Reticulocytes (*10^3^/µl)	0.07		0.02		0.07		0.02		0.68
Reticulocytes %	1.29		0.50		1.36		0.40		0.34
Protein C %	92.11		7.96		92.33		8.31		0.84
Protein %	111.11		13.13		114.56		1.38		0.09
**Chemical parameters**									
Glucose (mg/dl)	75.56		10.82		76.78		4.44		0.72
Hb Glycate (IFCC)	29.67		2.69		29.44		2.51		0.63
Urea (mg/dl)	36.89		6.77		37.11		6.33		0.97
Creatinine (mg/dl)	0.99		0.06		0.90		0.31		0.48
Uric acid (mg/dl)	5.39		0.64		5.27		0.69		0.63
Cholesterol (mg/dl)	156.89		16.54		153.78		17.16		0.15
HDL (mg/dl)	52.56		5.96		51.00		6.38		0.08
LDL (mg/dl)	95.44		11.25		93.67		11.70		0.21
Triglycerides (mg/dl)	63.67		16.88		65.44		21.37		0.71
Bilirubin T (mg/dl)	1.27		0.41		1.30		0.36		0.57
Bilirubin D (mg/dl)	0.25		0.06		0.22		0.06		0.36
Proteins T (g/dl)	7.80		0.34		7.70		0.26		0.19
Lactic acid	2.31		1.48		1.68		0.58		0.30
GOT (U/L)	30.67		6.63		32.89		9.96		0.74
GPT (U/L)	18.33		3.87		20.56		6.00		0.19
GGT (U/L)	19.00		4.74		18.44		5.03		0.38
CK (U/L)	426.67		300.11		428.33		244.63		> 0.99
Troponin (ng/L)	40.00		49.68		48.00		49.21		0.98
Amylase (U/L)	73.00		28.06		70.11		27.94		0.20
Sodium (mEq/L)	140.33		1.12		141.00		0.87		0.16
Potassium (mEq/L)	3.41		0.18		3.57		0.20		0.06
Phosphorus (mg/dl)	3.56		0.53		3.70		0.27		0.64
Iron (µg/dl)	82.33		28.20		94.44		31.80		0.43
Transferrin (mg/dl)	260.89		20.47		257.89		23.61		0.30
% Transferrin saturation	23.00		8.43		26.44		9.32		0.43
**Ferritin (ng/ml)**	89.00		27.04		75.67		20.30		**0.02**
**Hormones**									
TSH (µIU/ml)	2.26		0.90		2.23		1.13		> 0.99
**Testosterone (ng/dl)**	7.46		2.14		6.09		1.81		**0.01**
Cortisol (µg/dl)	14.29		3.30		15.11		4.10		0.55
**T/C ratio**	0.55		0.18		0.42		0.12		**0.01**
GH (ng/ml)	0.59		1.00		0.81		1.45		0.84
IGF1 (ng/ml)	313.39		58.62		322.56		53.49		0.20
peptide C (ng/ml)	1.23		0.32		1.16		0.32		0.44
Insulin (µIU/ml)	5.17		2.39		3.88		1.01		0.15
LH (mIU/ml)	3.60		1.47		3.18		1.10		0.48
FSH (mIU/ml)	2.18		0.80		1.97		0.54		0.19
**E2 (pg/ml)**	34.56		9.06		25.38		6.14		**0.02**
Progesterone (ng/ml)	0.27		0.11		0.33		0.17		0.50
**Cytokines and chemokines**									
CCL2 (pg/ml)	188.78		31.36		196.78		39.98		0.21
**IL-18 (pg/ml)**	210.00		68.77		179.11		52.15		**0.02**
IL-1ra (pg/ml)	219.11		86.95		191.67		41.02		0.27
**IL-2ra (pg/ml)**	1829.33		651.72		1479.56		596.64		**0.01**

### Monocyte phenotyping

**Fig. 3:**
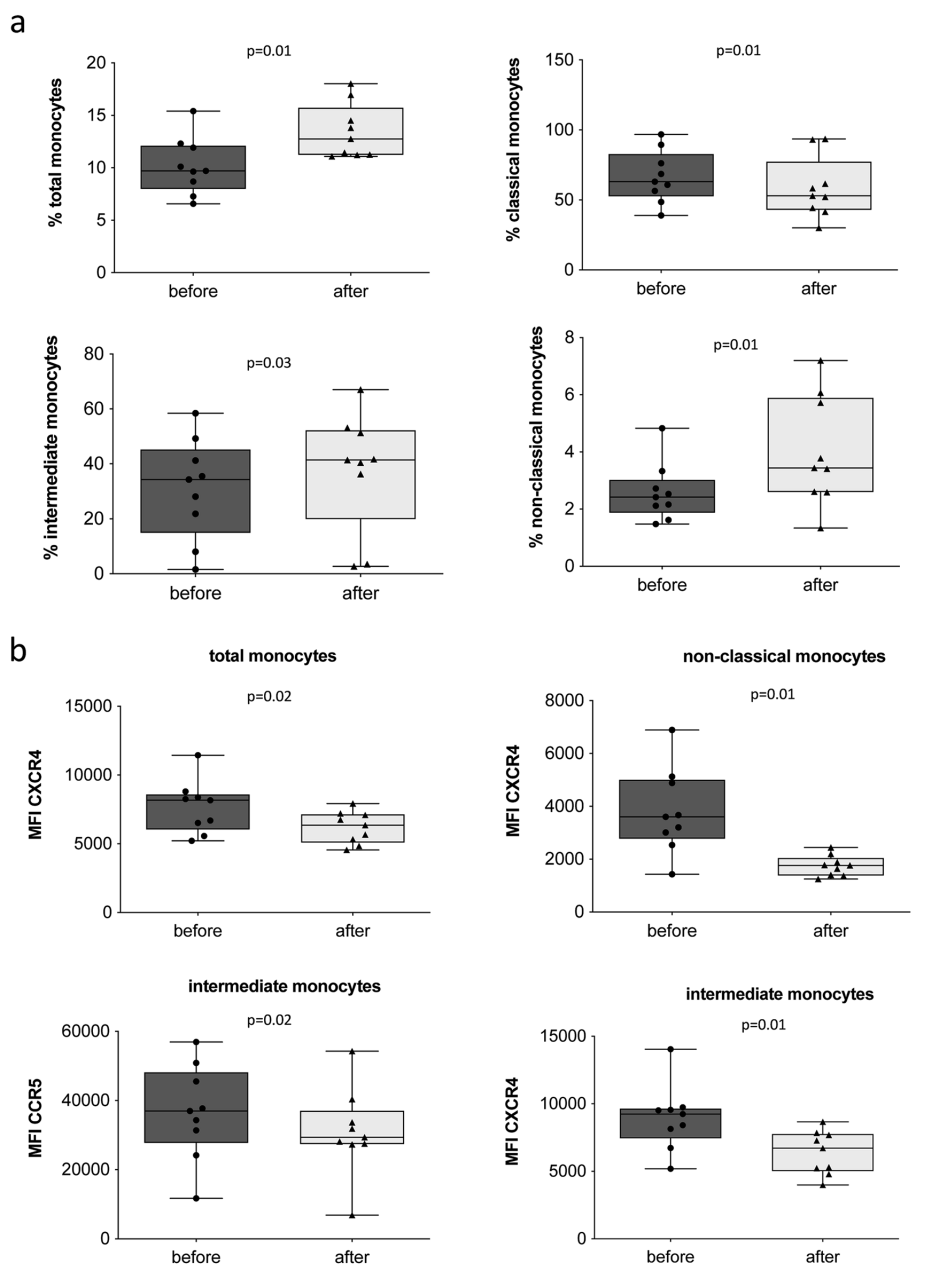
**Analysis of monocyte phenotype. (a)** Percentage of total monocytes among PBMCs, and percentage of classical, intermediate and non-classical monocytes among total PBMCs. **(b)** MFI of CXCR4 and CCR5 on total, classical, intermediate and non-classical monocytes from non-professional soccer players before and after five one-a-day sessions of WBC. Figures showing the single values in a box and whiskers plot. PBMCs indicates Peripheral blood mononuclear cells; MFI, Mean fluorescence Intensity; CXCR4, C-X-C chemokine receptor type 4; CCR5, C-C chemokine receptor type 5; WBC, whole-body cryotherapy.

To evaluate if WBC-t impact the innate immune response, we evaluated the relative percentage of the main monocyte subsets (classical, intermediate, and non-classical) and the changes in the levels of two surface receptors crucial for the homing of monocytes in inflamed tissue, namely CXCR4 and CCR5. Among the NPSPs, we observed an increase of the percentage of total, intermediate and non-classical monocytes among PBMCs (Fig. 3a, upper left panel) while classical monocytes were reduced (Fig. 3a, upper left and right panel). Moreover, significantly reduced levels of CXCR4 (intermediate and non-classical) and CCR5 were observed in all subsets (Fig. 3b).

### Quantification of soluble markers and circulating mtDNA

**Fig. 4:**
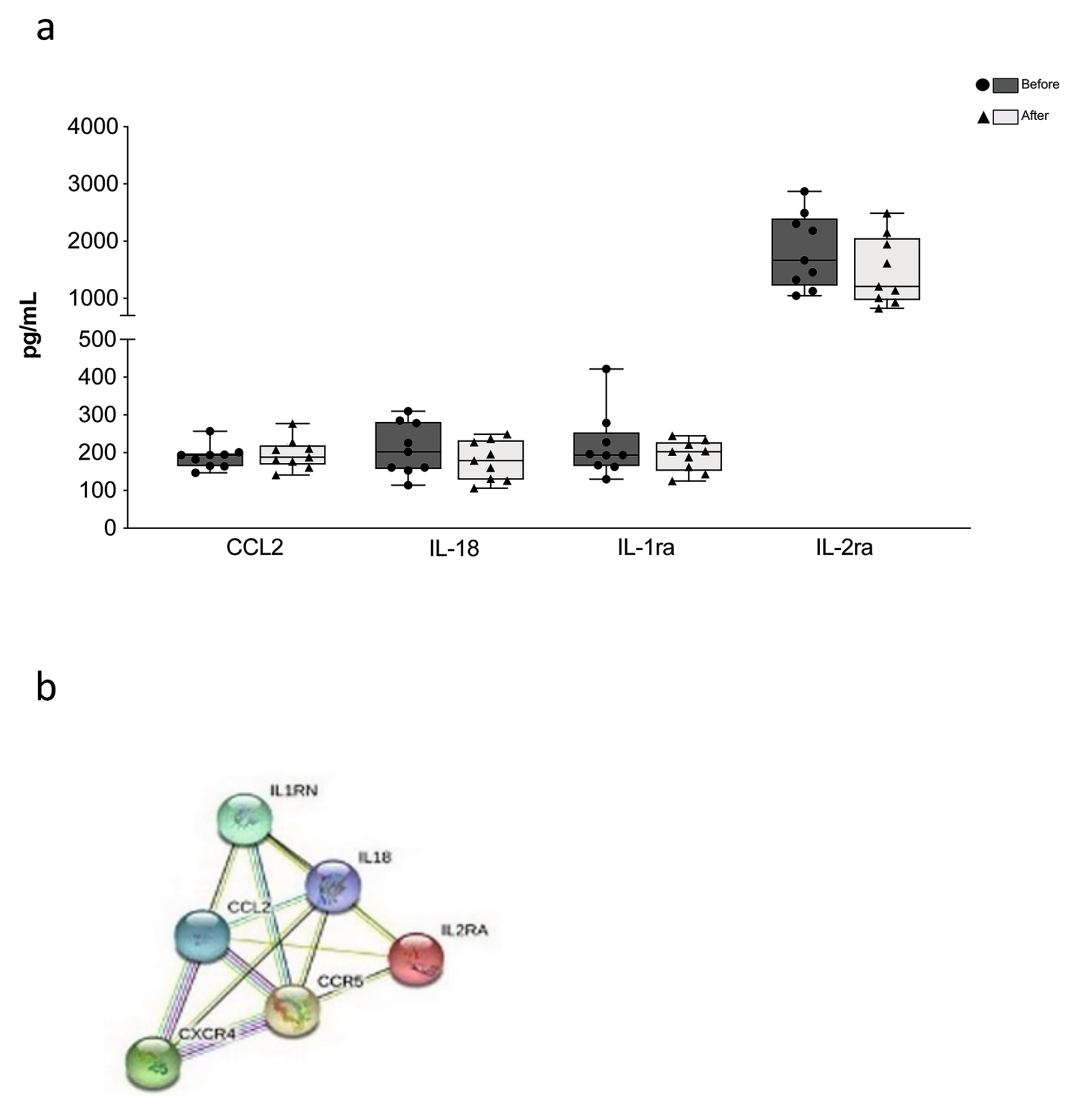
**Circulating cytokines and chemokines levels. (a)** Plasma levels of CCL2, IL-18, IL-1ra and IL-2ra before (black columns) and after (grey columns) five one-a-day sessions of WBC in non-professional soccer players. Figures showing the single values in a box and whiskers plot. * p < 0.05; ** p < 0.01; *** p < 0.001 **(b)** Plasma STRING analysis for the interactions among CCL2, IL-18, IL-1ra and IL-2ra. Blu and pink lines represent known interactions, from curated database and experimentally determined, respectively. Purple line indicates protein homology. CCL2 indicates C-C Motif Chemokine Ligand 2; IL-18, Interleukin 18; IL-1ra, interleukin-1 receptor antagonist; IL-2ra, interleukin 2 receptor subunit alpha; WBC, whole-body cryotherapy; STRING, Search Tool for the Retrieval of Interacting Genes/Proteins.

**Fig. 5:**
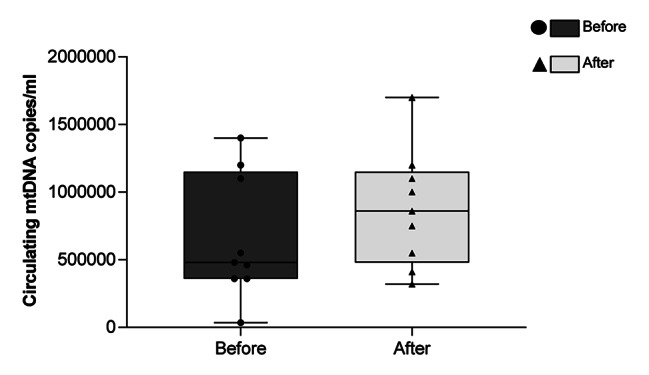
**Quantification of Circulating mtDNA levels.** Number of copies of mtDNA in plasma, before and after five one-a-day sessions of WBC, as measured by ddPCR. The figure showing the single values in a box and whiskers plot. mtDNA indicates mitochondrial DNA; WBC, whole-body cryotherapy; ddPCR, droplet digital PCR.

Concerning plasma parameters, we have quantified the four soluble factors that we previously found to be modulated by WBC-t in cyclists and runners. In soccer players, a decrease of IL-18 and IL-2RA, a tendency towards decreases of IL-1RA and a tendency towards an increase of CCL2 were registered, as shown in the Fig. 4a. Moreover, STRING analysis (https://string-db.org/, version 11.0) revealed that IL-18, IL1RN, IL2RA and CCL2 were strongly connected to each other (Fig. 4b).We finally determined the levels of circulating mtDNA, a molecule related to cell damage that can have a pro-inflammatory effect, and we did not observe changes before and after WBC-t (Fig. 5).

## Discussion

Long-term training and inadequate rest lead to biochemical changes, muscle damage, chronic fatigue, increased susceptibility to injury and inflammation. All these effects are reflected in a performance decline [11, 12]. Therefore, this study focused on the use of WBC as a recovery strategy for non-professional male soccer players, through the analysis of an exhaustive collection of hematochemical and hormonal parameters. WBC-t seems do not affect the frequency and absolute count of different types of leukocytes while was associated with variations of some parameters: we observed reduced levels of ferritin (normal range in male 20–300 ng/ml), MCH (normal range 26–32 pg), and MPV (normal range 9.7–12.8 fl.), following WBC-t. Moreover, a slight decrease of Hb concentration following WBC-t was observed.

Data regarding hematological variations associated with WBC-t are controversial. Silva et al. and Heisterberg et al. reported a significant increase in Hb levels and a decrease of PV after three months of intense training [13]. These modifications could be explained by a concept called “sports anemia”, which links the increase in Hb concentration to the stimulation of erythrocytosis induced by a period of intense exercise. For this reason, sports anemia is considered one of the first signs of overtraining. This mechanism is usually counteracted by an increase of PV [14]. However, Requena et al. also reported a significant increase in both Hb and Ht levels in athletes following a period of six-weeks off, characterized by a great reduction in training volume and intensity [15]. It has been suggested that Hb and Ht can be measured at the same time to monitor the initiation of a state of over-fatigued. In soccer, improved performance has been proven to be related to significant increases in both Hb and Ht concentrations at rest [13]. Hormones are largely analyzed to evaluate the performance of athletes because they are often used as biomarkers of anabolic status, training responses and motivation [16]. Therefore, evaluation of changes in anabolic hormones, such as testosterone, and the testosterone/cortisol (T/C) ratio is crucial for monitoring muscle recovery [1]. Testosterone is believed to be a primary anabolic hormone involved in protein synthesis, protecting from skeletal muscle degradation. In our study, we found a significant decrease of testosterone (normal range in male of 20–25 years old 5.25–20.7 ng/dl) and E2 (normal range in male 10–45 pg/ml). These two hormones are closely related because testosterone in excess is transformed into estradiol, which in turn acts as an inhibitor of testosterone production. Our group previously published hormonal variations among cyclists and runners following WBC-t, without any reduction in testosterone levels reported [10]. These contrasting findings may be explained by the different study designs; cyclists and runners only underwent three WBC-t sessions. Nevertheless, these data suggest a potential role of WBC-t as an important recovery strategy for athletes after a period of intense training and competitions. Saidi et al. showed that long-term training and competitions can induce a reduction in testosterone concentrations [17]. We cannot exclude that, at least in part, the observed reduction of testosterone could be due to the training. Filaire et al. however reported a substantial increase in cortisol at the end of the soccer season, suggesting that the high intensity of training could induce a prolonged increase in diurnal cortisol secretion [18]. Hormonal changes following long-term soccer training are believed to be linked to physical performance and reflect not only the training volume and intensity, but also recovery strategies [1]. Inadequate recovery after a sport season can promote a state of fatigue and increase the risk of injury. Several studies have suggested that a decline in T/C ratio could lead to overtraining syndrome characterized by exhaustion, lethargy, tiredness, and negative physical performance changes [19, 20].

Regarding cytokines and chemokines involved in inflammatory processes, the results from our study show a slight decrease in the expression of IL2RA, IL1RN and IL-18. Our previous studies of WBC-t on cyclists and runners reported increases in these cytokines and chemokines [10]. However, a trend in the increase of CCL2, which acts as a potent monocyte attractant [21], was observed both in this study on soccer players and in our previous study on cyclists and runners. WBC inducing CCL2 increase could have an important impact in tissue repair through the chemoattraction of monocytes into the tissue. Moreover, IL-18 is involved in inflammasome activation and increases after regular physical activity in skeletal muscle [22] but not in plasma [24]. Hence, these modifications suggest that WBC potentially enhances immunosurveillance [10]. Furthermore, the differences observed between soccer players and cyclists and runners could be due to training protocols, treatment duration and different types and levels of physical activity. As highlighted by STRING analysis, IL-18, IL1RN, IL2RA and CCL2 were strongly connected, indicating a role for WBC-t in modulating the production of cytokine involved in inflammation and tissue repair [23].

In NPSP, we observed an increase in the percentage of total, intermediate and non-classical monocytes after WBC-t, whilst the percentage of classical monocytes reduced. Our hypothesis is that classical monocytes, following WBC-t, are the first to be redistributed in surrounding tissue. Despite an overall increase in the percentage of intermediate and non-classical monocytes, CXCR4 expression was reduced. Since CXCR4 is a surface receptor that promotes migration and survival of monocytes, it is likely that monocytes with high expression of CXCR4 had already migrated to surrounding tissue These results mirror only in part those found in cyclists and runners [10], in which the percentage of total monocytes did not change. Moreover, cyclists showed a decrease in intermediate and non-classical monocytes, and runners showed a decrease in non-classical monocytes. We hypothesize that the differences in monocyte redistribution may be due to the different durations of WBC-t treatment (3 one-a-day sessions in the previous study) and the intensity of the individual sport, affecting how quickly innate immunity is activated.

Circulating-free mitochondrial DNA (cf-mtDNA) could be release by damaged cells from injured tissues and acts as a damage-associated molecular pattern inducing inflammation. An increase of cf-mtDNA is associated with different physio-pathological conditions (such as HIV, Multiple Sclerosis, aging) [23, 25, 26]. No specific studies regarding mtDNA in soccer players have been reported so far. However, it has been shown that blood cell free DNA (which include both nuclear and mitochondrial DNA) undergoes a strong increase after a training session in professional soccer players, with a good correlation with the players’ exercise load [27]. Cell-free DNA increases not only in soccer players, but also after aerobic running, cycling and strength training, among others, suggesting that release of DNA in the bloodstream is always present after exercise, and can represent a better blood-based biomarker of exercise than established molecules such as CRP or IL-6 [28–30]. As we did not observe any increase of mtDNA in our cohort, we are tempted to speculate that WBC-t can strongly reduce the release of this molecule in the blood. This observation is particularly relevant as mtDNA, rather than nuclear DNA, has a pro inflammatory effect [31]. Several studies have reported a decrease of circulating mtDNA induced by regular exercise, confirming this protective, anti-inflammatory effect of exercise [32]. Conversely, intense exercise seems to have the opposite effect [33]. As the released mtDNA triggers the development of severe tissue injury [23, 29, 30], the possibility to keep its levels low with WBC-t can be or great help for reducing post-traumatic systemic inflammatory response of players [34].

## Conclusion

WBC-t seems to induce changes of the innate components of the immune system (hormone profile, hematologic parameters, and serum chemistry) in NPSP, suggesting it has not only a beneficial anti-inflammatory effect but also an important role in tissue repair. Therefore, these results could open a new field of research, and new possible treatment strategies to minimize the inflammatory status and to improve performance. Therefore, the regular use of WBC could be considered an effective treatment to counteract inflammation, to prevent injuries and to improve the recovery from traumas in soccer players.

## Electronic supplementary material

Below is the link to the electronic supplementary material.

Supplementary Material 1

## Data Availability

The datasets generated and/or analysed during the current study are available from the corresponding author on reasonable request.
