# Supplementary material for: Innate immunity changes in soccer players after whole-body cryotherapy
Source: BMC Sports Sci Med Rehabil. 2022 Oct 25;14:185. doi: 10.1186/s13102-022-00578-z (PMC9594892; doi:10.1186/s13102-022-00578-z)
Supplement: Supplementary file 1 — Supplementary Material 1 [file 13102_2022_578_MOESM1_ESM.docx]

|  |  | | | | |  | | | | | | | | | | |
| --- | --- | --- | --- | --- | --- | --- | --- | --- | --- | --- | --- | --- | --- | --- | --- | --- |
|  |  |  |  |  |  |  |  |  |  |  |  |  |  |  |  |  |
|  | **Subject 1** | | | **Subject 2** | | **Subject 3** | | **Subject 4** | | **Subject 5** | | **Subject 6** | | **Subject 7** | | |
|  | **T0** | **T1** | | **T0** | **T1** | **T0** | **T1** | **T0** | **T1** | **T0** | **T1** | **T0** | **T1** | **T0** | **T1** | |
| **Hematological parameters** | | | | | | | | | | | | | | | | |
| White blood cells (*10^3^/µl) | 6.79 | | 7.90 | 6.30 | 6.40 | 7.74 | 6.40 | 5.78 | 6.30 | 6.62 | 5.60 | 5.69 | 6.80 | 5.82 | 8.50 | |
| Red blood cells (*10^3^/µl) | 5.51 | | 5.43 | 5.18 | 5.04 | 5.17 | 5.07 | 4.85 | 4.88 | 5.28 | 5.34 | 5.22 | 5.05 | 5.22 | 4.82 | |
| Hemoglobin (g/dl) | 16.20 | | 16.00 | 15.50 | 15.30 | 15.40 | 15.10 | 15.50 | 15.90 | 14.90 | 15.20 | 15.60 | 15.30 | 15.20 | 14.60 | |
| Hematocrit (%) | 89.40 | | 48.90 | 47.30 | 45.50 | 46.40 | 45.80 | 46.50 | 46.70 | 46.20 | 46.40 | 46.70 | 46.00 | 45.80 | 44.00 | |
| MCV (fl) | 89.40 | | 90.10 | 91.40 | 90.40 | 89.70 | 90.30 | 95.80 | 95.70 | 87.50 | 86.80 | 89.50 | 91.00 | 87.80 | 91.30 | |
| **MCH (pg)** | 29.40 | | 29.60 | 29.80 | 30.40 | 29.80 | 29.70 | 31.90 | 32.60 | 28.30 | 28.50 | 29.90 | 30.30 | 29.80 | 30.20 | |
| MCHC (g/dl) | 32.80 | | 32.80 | 32.60 | 33.60 | 33.20 | 32.90 | 33.30 | 34.10 | 32.20 | 32.80 | 33.40 | 33.30 | 33.90 | 33.10 | |
| RDW (cv%) | 14.00 | | 13.80 | 15.00 | 14.80 | 13.60 | 13.90 | 13.20 | 13.40 | 14.90 | 14.60 | 13.60 | 13.30 | 12.80 | 13.00 | |
| Platelets (*10^3^/µl) | 217.00 | | 209.00 | 182.00 | 186.00 | 222.00 | 239.00 | 193.00 | 200.00 | 217.00 | 246.00 | 189.00 | 183.00 | 195.00 | 190.00 | |
| **MPV (fl)** | 9.20 | | 8.90 | 8.40 | 8.50 | 9.10 | 8.70 | 9.30 | 9.10 | 8.70 | 8.30 | 9.00 | 8.80 | 9.00 | 9.20 | |
| Neutrophils % | 43.10 | | 42.30 | 44.30 | 48.20 | 45.50 | 47.40 | 56.60 | 52.70 | 59.40 | 52.40 | 48.50 | 52.90 | 54.00 | 60.80 | |
| Lymphocytes % | 42.50 | | 42.80 | 44.80 | 39.20 | 39.30 | 39.60 | 35.00 | 38.90 | 29.20 | 34.50 | 38.60 | 34.90 | 34.70 | 29.50 | |
| Monocytes % | 9.70 | | 9.30 | 7.60 | 9.00 | 9.20 | 7.90 | 6.90 | 6.70 | 9.30 | 9.10 | 7.20 | 6.60 | 5.50 | 5.10 | |
| Eosinophils % | 4.00 | | 4.90 | 2.70 | 2.70 | 5.70 | 5.00 | 1.10 | 1.30 | 1.80 | 3.40 | 4.80 | 4.90 | 5.30 | 4.20 | |
| Basophils % | 0.70 | | 0.70 | 0.60 | 0.60 | 0.40 | 0.10 | 0.40 | 0.40 | 0.30 | 0.40 | 0.90 | 0.70 | 0.60 | 0.40 | |
| Neutrofili (*10^3^/µl) | 2.93 | | 3.34 | 2.79 | 3.08 | 3.52 | 3.03 | 3.23 | 3.32 | 3.93 | 2.93 | 2.76 | 3.60 | 3.14 | 5.17 | |
| Lymphocytes (*10^3^/µl) | 2.89 | | 3.38 | 2.82 | 2.53 | 3.04 | 2.53 | 2.00 | 2.45 | 1.93 | 1.93 | 2.20 | 2.37 | 2.02 | 2.51 | |
| Monocytes (*10^3^/µl) | 0.66 | | 0.73 | 0.48 | 0.58 | 0.71 | 0.51 | 0.39 | 0.42 | 0.62 | 0.51 | 0.41 | 0.45 | 0.32 | 0.43 | |
| Basophils (*10^3^/µl) | 0.05 | | 0.06 | 0.04 | 0.04 | 0.03 | 0.01 | 0.02 | 0.03 | 0.02 | 0.02 | 0.05 | 0.05 | 0.03 | 0.03 | |
| Reticulocytes (*10^3^/µl) | 0.073 | | 0.057 | 0.095 | 0.096 | 0.056 | 0.090 | 0.12 | 0.095 | 0.045 | 0.049 | 0.055 | 0.52 | 0.054 | 0.052 | |
| Reticulocytes % | 1.32 | | 1.05 | 1.84 | 1.91 | 1.09 | 1.71 | 2.37 | 1.95 | 0.86 | 0.92 | 1.05 | 1.02 | 1.04 | 1.09 | |
| Protein C % | 105.00 | | 105.00 | 89.00 | 81.00 | 92.00 | 92.00 | 102.00 | 95.00 | 80.00 | 82.00 | 91.00 | 96.00 | 96.00 | 97.00 | |
| Protein % | 130.00 | | 134.00 | 104.00 | 96.00 | 102.00 | 102.00 | 105.00 | 118.00 | 102.00 | 118.00 | 100.00 | 102.00 | 114.00 | 115.00 | |
| **Chemical parameters** | | | | | | | | | | | | | | | | |
| Glucose (mg/dl) | 73.00 | | 80.00 | 81.00 | 75.00 | 91.00 | 86.00 | 66.00 | 73.00 | 85.00 | 74.00 | 77.00 | 76.00 | 84.00 | 72.00 | |
| Hb Glycate (IFCC) | 31.00 | | 30.00 | 26.00 | 26.00 | 30.00 | 31.00 | 31.00 | 31.00 | 32.00 | 31.00 | 25.00 | 25.00 | 32.00 | 32.00 | |
| Urea (mg/dl) | 37.00 | | 37.00 | 33.00 | 46.00 | 35.00 | 28.00 | 48.00 | 34.00 | 38.00 | 31.00 | 23.00 | 33.00 | 39.00 | 46.00 | |
| Creatinine (mg/dl) | 1.01 | | 1.04 | 1.02 | 1.08 | 0.96 | 0.93 | 0.97 | 0.97 | 1.00 | 1.02 | 0.88 | 0.95 | 0.98 | 0.99 | |
| Uric acid (mg/dl) | 5.30 | | 5.90 | 4.20 | 4.60 | 4.90 | 4.60 | 5.20 | 4.90 | 6.00 | 6.60 | 5.90 | 5.80 | 5.10 | 4.90 | |
| Cholesterol (mg/dl) | 169.00 | | 174.00 | 128.00 | 127.00 | 162.00 | 157.00 | 154.00 | 156.00 | 154.00 | 152.00 | 189.00 | 183.00 | 158.00 | 148.00 | |
| HDL (mg/dl) | 57.00 | | 54.00 | 50.00 | 49.00 | 47.00 | 43.00 | 46.00 | 48.00 | 52.00 | 52.00 | 66.00 | 66.00 | 51.00 | 50.00 | |
| LDL (mg/dl) | 100.00 | | 106.00 | 75.00 | 73.00 | 105.00 | 102.00 | 100.00 | 101.00 | 94.00 | 91.00 | 112.00 | 107.00 | 100.00 | 94.00 | |
| Triglycerides (mg/dl) | 97.00 | | 109.00 | 44.00 | 64.00 | 56.00 | 61.00 | 53.00 | 60.00 | 50.00 | 56.00 | 53.00 | 46.00 | 76.00 | 49.00 | |
| Bilirubin T (mg/dl) | 0.58 | | 1.63 | 1.02 | 1.19 | 1.09 | 1.56 | 1.55 | 1.37 | 0.00 | 0.88 | 1.77 | 1.33 | 1.78 | 1.87 | |
| Bilirubin D (mg/dl) | 0.14 | | 0.27 | 0.25 | 0.22 | 0.21 | 0.25 | 0.29 | 0.20 | 0.20 | 0.17 | 0.29 | 0.24 | 0.33 | 0.33 | |
| Proteins T (g/dl) | 7.50 | | 7.50 | 7.40 | 7.30 | 7.30 | 7.30 | 7.70 | 7.90 | 8.20 | 7.80 | 8.10 | 7.90 | 7.80 | 7.80 | |
| Lactic acid | 2.50 | | 2.50 | 5.20 | 2.20 | 2.60 | 1.60 | 1.00 | 1.00 | 3.90 | 1.60 | 2.40 | 1.40 | 1.40 | 2.40 | |
| GOT (U/L) | 24.00 | | 28.00 | 31.00 | 49.00 | 26.00 | 30.00 | 38.00 | 28.00 | 31.00 | 22.00 | 25.00 | 34.00 | 26.00 | 33.00 | |
| GPT (U/L) | 13.00 | | 16.00 | 20.00 | 31.00 | 17.00 | 17.00 | 24.00 | 22.00 | 18.00 | 16.00 | 15.00 | 17.00 | 19.00 | 20.00 | |
| GGT (U/L) | 15.00 | | 15.00 | 13.00 | 12.00 | 24.00 | 25.00 | 28.00 | 26.00 | 20.00 | 18.00 | 15.00 | 13.00 | 19.00 | 19.00 | |
| CK (U/L) | 93.00 | | 135.00 | 274.00 | 807.00 | 442.00 | 439.00 | 1103.00 | 483.00 | 444.00 | 177.00 | 298.00 | 574.00 | 131.00 | 388.00 | |
| Troponin (ng/L) | 2.00 | | 2.00 | 9.00 | 17.00 | 96.00 | 66.00 | 9.00 | 4.00 | 14.00 | 6.00 | 36.00 | 140.00 | 8.00 | 82.00 | |
| Amylase (U/L) | 111.00 | | 100.00 | 60.00 | 58.00 | 73.00 | 63.00 | 126.00 | 129.00 | 54.00 | 50.00 | 72.00 | 66.00 | 55.00 | 60.00 | |
| Sodium (mEq/L) | 141.00 | | 142.00 | 141.00 | 140.00 | 141.00 | 141.00 | 138.00 | 140.00 | 140.00 | 141.00 | 140.00 | 142.00 | 140.00 | 141.00 | |
| Potassium (mEq/L) | 3.40 | | 3.30 | 3.20 | 3.20 | 3.30 | 3.80 | 3.30 | 3.60 | 3.30 | 3.60 | 3.30 | 3.50 | 3.70 | 3.70 | |
| Phosphorus (mg/dl) | 3.80 | | 3.80 | 3.90 | 3.90 | 4.30 | 3.70 | 2.40 | 3.30 | 3.60 | 3.90 | 3.30 | 3.80 | 3.30 | 3.70 | |
| Iron (µg/dl) | 61.00 | | 155.00 | 82.00 | 100.00 | 81.00 | 81.00 | 89.00 | 76.00 | 64.00 | 85.00 | 132.00 | 116.00 | 114.00 | 81.00 | |
| Transferrin (mg/dl) | 261.00 | | 250.00 | 270.00 | 276.00 | 280.00 | 277.00 | 241.00 | 244.00 | 255.00 | 244.00 | 254.00 | 251.00 | 248.00 | 250.00 | |
| % Transferrin saturation | 17.00 | | 44.00 | 22.00 | 26.00 | 21.00 | 28.00 | 26.00 | 22.00 | 18.00 | 25.00 | 37.00 | 33.00 | 33.00 | 23.00 | |
| **Ferritin (ng/ml)** | 96.00 | | 96.00 | 76.00 | 70.00 | 47.00 | 48.00 | 143.00 | 114.00 | 92.00 | 87.00 | 62.00 | 57.00 | 87.00 | 74.00 | |
| **Hormones** | | | | | | | | | | | | | | | | |
| TSH (µIU/ml) | 1.82 | | 1.85 | 2.93 | 2.71 | 2.30 | 1.65 | 1.75 | 3.76 | 2.69 | 1.04 | 0.72 | 1.04 | 3.87 | 4.24 | |
| **Testosterone (ng/dl)** | 7.70 | | 6.00 | 6.30 | 5.70 | 7.50 | 6.30 | 7.70 | 7.40 | 6.90 | 6.00 | 7.00 | 4.50 | 4.70 | 3.30 |  |
| Cortisol (µg/dl) | 11.40 | | 16.20 | 15.70 | 15.80 | 8.70 | 10.00 | 12.10 | 14.50 | 17.10 | 21.80 | 12.70 | 9.30 | 17.30 | 12.20 |  |
| **T/C ratio** | 0.68 | | 0.37 | 0.40 | 0.36 | 0.86 | 0.63 | 0.64 | 0.51 | 0.40 | 0.28 | 0.55 | 0.48 | 0.27 | 0.27 |  |
| GH (ng/ml) | 0.02 | | 0.03 | 0.10 | 0.09 | 3.18 | 1.20 | 0.52 | 0.10 | 0.77 | 1.23 | 0.10 | 0.12 | 0.23 | 0.04 |  |
| IGF1 (ng/ml) | 229.60 | | 248.70 | 371.50 | 365.70 | 328.00 | 317.00 | 301.70 | 295.50 | 406.70 | 428.70 | 314.00 | 321.20 | 354.70 | 349.40 |  |
| peptide C (ng/ml) | 1.37 | | 0.99 | 1.22 | 1.05 | 1.85 | 1.52 | 1.31 | 1.40 | 1.33 | 1.01 | 1.25 | 0.96 | 1.15 | 1.47 |  |
| Insulin (µIU/ml) | 3.30 | | 2.90 | 10.70 | 4.60 | 6.90 | 5.00 | 4.00 | 4.00 | 4.30 | 2.70 | 5.20 | 3.70 | 5.20 | 4.60 |  |
| LH (mIU/ml) | 3.60 | | 3.10 | 3.70 | 4.40 | 4.40 | 3.80 | 5.30 | 4.00 | 2.00 | 2.10 | 2.10 | 1.60 | 1.80 | 2.00 |  |
| FSH (mIU/ml) | 1.70 | | 1.50 | 2.50 | 2.50 | 2.10 | 1.90 | 2.70 | 2.40 | 2.00 | 2.00 | 1.90 | 1.50 | 1.10 | 1.10 |  |
| **E2 (pg/ml)** | 31.00 | | 27.00 | 37.00 | 28.00 | 23.00 | 29.00 | 47.00 | 35.00 | 37.00 | 21.00 | 35.00 | 27.00 | 20.00 | 15.00 |  |
| Progesterone (ng/ml) | 0.20 | | 0.60 | 0.30 | 0.30 | 0.10 | 0.10 | 0.20 | 0.20 | 0.30 | 0.50 | 0.20 | 0.20 | 0.30 | 0.30 |  |
| **Cytokines and chemokines** | | | | | | | | | | | | | | | | |
| CCL2 (pg/ml) | 194.00 | | 177.00 | 164.00 | 161.00 | 257.00 | 277.00 | 165.00 | 181.00 | 194.00 | 211.00 | 201.00 | 188.00 | 195.00 | 227.00 | |
| **IL-18 (pg/ml)** | 153.00 | | 160.00 | 114.00 | 106.00 | 285.00 | 196.00 | 278.00 | 237.00 | 226.00 | 228.00 | 202.00 | 179.00 | 310.00 | 249.00 | |
| IL-1ra (pg/ml) | 167.00 | | 162.00 | 228.00 | 188.00 | 422.00 | 245.00 | 196.00 | 143.00 | 194.00 | 233.00 | 193.00 | 222.00 | 279.00 | 204.00 | |
| **IL-2ra (pg/ml)** | 1046.00 | | 1008.00 | 1457.00 | 1141.00 | 1323.00 | 827.00 | 2869.00 | 2490.00 | 2302.00 | 1945.00 | 2491.00 | 2153.00 | 2184.00 | 1614.00 | |

|  | **Subject 8** | | **Subject 9** | |
| --- | --- | --- | --- | --- |
|  | **T0** | **T1** | **T0** | **T1** |
| **Hematological parameters** | | | | |
| White blood cells (*10^3^/µl) | 9.31 | 6.80 | 6.52 | 5.47 |
| Red blood cells (*10^3^/µl) | 5.14 | 5.15 | 5.64 | 5.66 |
| Hemoglobin (g/dl) | 14.80 | 15.00 | 15.80 | 16.20 |
| Hematocrit (%) | 45.00 | 45.50 | 48.40 | 48.50 |
| MCV (fl) | 87.60 | 88.30 | 85.90 | 85.70 |
| **MCH (pg)** | 28.80 | 29.20 | 28.10 | 28.60 |
| MCHC (g/dl) | 32.90 | 33.00 | 32.70 | 33.40 |
| RDW (cv%) | 14.80 | 14.20 | 14.20 | 14.30 |
| Platelets (*10^3^/µl) | 171.00 | 166.00 | 144.00 | 103.00 |
| **MPV (fl)** | 9.70 | 8.90 | 9.00 | 8.40 |
| Neutrophils % | 66.40 | 68.70 | 44.50 | 46.90 |
| Lymphocytes % | 24.90 | 22.30 | 45.30 | 42.30 |
| Monocytes % | 7.20 | 7.30 | 7.00 | 7.60 |
| Eosinophils % | 0.90 | 0.90 | 2.40 | 2.80 |
| Basophils % | 0.60 | 0.80 | 0.90 | 0.50 |
| Neutrofili (*10^3^/µl) | 6.18 | 4.67 | 2.90 | 2.57 |
| Lymphocytes (*10^3^/µl) | 2.32 | 1.52 | 2.95 | 2.31 |
| Monocytes (*10^3^/µl) | 0.67 | 0.50 | 0.46 | 0.42 |
| Basophils (*10^3^/µl) | 0.06 | 0.05 | 0.06 | 0.03 |
| Reticulocytes (*10^3^/µl) | 0.055 | 0.071 | 0.054 | 0.071 |
| Reticulocytes % | 1.07 | 1.37 | 0.95 | 1.25 |
| Protein C % | 84.00 | 84.00 | 90.00 | 99.00 |
| Protein % | 136.00 | 138.00 | 107.00 | 108.00 |
| **Chemical parameters** | | | | |
| Glucose (mg/dl) | 59.00 | 75.00 | 64.00 | 80.00 |
| Hb Glycate (IFCC) | 28.00 | 28.00 | 32.00 | 31.00 |
| Urea (mg/dl) | 42.00 | 38.00 | 37.00 | 41.00 |
| Creatinine (mg/dl) | 1.08 | 0.083 | 1.04 | 1.06 |
| Uric acid (mg/dl) | 6.30 | 5.20 | 5.60 | 4.90 |
| Cholesterol (mg/dl) | 147.00 | 136.00 | 151.00 | 151.00 |
| HDL (mg/dl) | 52.00 | 49.00 | 52.00 | 48.00 |
| LDL (mg/dl) | 85.00 | 80.00 | 88.00 | 89.00 |
| Triglycerides (mg/dl) | 96.00 | 51.00 | 75.00 | 93.00 |
| Bilirubin T (mg/dl) | 1.10 | 1.08 | 1.29 | 0.75 |
| Bilirubin D (mg/dl) | 0.25 | 0.20 | 0.26 | 0.14 |
| Proteins T (g/dl) | 8.10 | 7.90 | 8.10 | 7.90 |
| Lactic acid | 1.00 | 1.50 | 0.80 | 0.90 |
| GOT (U/L) | 44.00 | 49.00 | 31.00 | 23.00 |
| GPT (U/L) | 24.00 | 30.00 | 15.00 | 16.00 |
| GGT (U/L) | 20.00 | 22.00 | 17.00 | 16.00 |
| CK (U/L) | 570.00 | 709.00 | 485.00 | 143.00 |
| Troponin (ng/L) | 138.00 | 66.00 | 10.00 | 3.00 |
| Amylase (U/L) | 67.00 | 69.00 | 39.00 | 36.00 |
| Sodium (mEq/L) | 142.00 | 142.00 | 140.00 | 140.00 |
| Potassium (mEq/L) | 3.60 | 3.70 | 3.60 | 3.70 |
| Phosphorus (mg/dl) | 3.70 | 3.20 | 3.70 | 4.00 |
| Iron (µg/dl) | 37.00 | 39.00 | 81.00 | 89.00 |
| Transferrin (mg/dl) | 302.00 | 304.00 | 237.00 | 225.00 |
| % Transferrin saturation | 9.00 | 9.00 | 24.00 | 28.00 |
| **Ferritin (ng/ml)** | 97.00 | 69.00 | 101.00 | 66.00 |
| **Hormones** | | | | |
| TSH (µIU/ml) | 2.58 | 1.87 | 1.65 | 1.95 |
| **Testosterone (ng/dl)** | 12.60 | 9.80 | 6.70 | 5.80 |
| Cortisol (µg/dl) | 18.90 | 18.90 | 14.70 | 17.30 |
| **T/C ratio** | 0.67 | 0.52 | 0.46 | 0.34 |
| GH (ng/ml) | 0.29 | 4.45 | 0.10 | 0.07 |
| IGF1 (ng/ml) | 255.70 | 272.80 | 258.60 | 304.00 |
| peptide C (ng/ml) | 0.74 | 0.57 | 0.84 | 1.46 |
| Insulin (µIU/ml) | 3.90 | 2.40 | 3.00 | 5.00 |
| LH (mIU/ml) | 6.00 | 3.00 | 3.50 | 4.60 |
| FSH (mIU/ml) | 3.90 | 2.80 | 1.70 | 2.00 |
| **E2 (pg/ml)** | 46.00 | 21.00 | 35.00 | 10.00 |
| Progesterone (ng/ml) | 0.50 | 0.30 | 0.30 | 0.50 |
| **Cytokines and chemokines** | | | | |
| CCL2 (pg/ml) | 182.00 | 208.00 | 147.00 | 141.00 |
| **IL-18 (pg/ml)** | 161.00 | 126.00 | 161.00 | 131.00 |
| IL-1ra (pg/ml) | 163.00 | 203.00 | 130.00 | 125.00 |
| **IL-2ra (pg/ml)** | 1666.00 | 1208.00 | 1126.00 | 930.00 |

**Supplementary table 1.** **Single** **hematochemical parameters**. Evaluation of hematological and chemical parameters, hormone profile, cytokines and chemokines level before (T0) and after (T1) five once-a-day sessions of WBC-t. The table shows the respecting single value for each subject. C indicates cortisol; CCL2, C-C Motif Chemokine Ligand 2; CK, creatine kinase; E2, estradiol; FSH, follicle-stimulating hormone; GGT, gamma-glutamil transferase; GH, growth hormone; GOT, glutamic oxaloacetic transaminase; GPT, pyruvic glutamic transaminase; HDL, high-density lipoprotein; IGF1, insulin growth factor 1; IL-1ra, interleukin 1 Receptor Antagonist; IL-2ra, interleukin 1 Receptor Antagonist, IL-18, interleukin 18; LDL, low-density lipoprotein; LH, luteinizing hormone; MCH, mean corpuscular hemoglobin; MCHC, mean corpuscular hemoglobin concentration; MCV, mean corpuscular volume; MPV, mean platelet volume; RDW, red cell distribution width; T, testosterone; TSH, thyroid stimulating hormone.
